# The causal association between neutrophil counts and the risk of lung cancer: a Mendelian randomization study

**DOI:** 10.7150/jca.100884

**Published:** 2025-01-01

**Authors:** Yunzhao Ren, Zhaojun Lu, Xiaoxue Liu

**Affiliations:** 1 School of Public Health, Zhejiang University School of Medicine, 866 Yuhangtang Rd., Hangzhou, Zhejiang 310058, China.; 2 HangZhou Center for Disease Control and Prevention, 568 Mingshi Road, Hangzhou, Zhejiang 310021, China.; 3 Global Health Research Division, Public Health Research Center and Department of Public Health and Preventive Medicine, Wuxi School of Medicine, Jiangnan University, Wuxi, Jiangsu 214122, China.

**Keywords:** Lung cancer, Neutrophil, Mendelian randomization, Causal association

## Abstract

An increased neutrophil level in the blood is considered a risk factor for lung cancer (LC). However, establishment of causality is hampered by the inconsistent findings of observational studies. This study aimed to explore the causal association between neutrophil counts (NC) and LC risk in two populations via two-sample mendelian randomization (MR) analysis. The inverse-variance weighted method was used to evaluate causality. Sensitivity analyses were conducted to examine the stability of the results. Bidirectional MR analysis was performed to check reverse causality, and a multivariable MR analysis was conducted to adjust for confounding factors. The results revealed a significant causal relationship between NC and LC (OR=1.027, 95% CI: 1.005-1.050, *P=*0.017) in the European population but not in the East Asian population (OR=1.223, 95% CI: 0.999-1.497; *P*=0.052). The sensitivity analysis confirmed the robustness of the results, and we excluded potential reverse causation. A multivariable analysis demonstrated that a significant genetic association (OR=1.044, 95% CI: 1.002-1.088, *P*=0.042) remained after controlling for smoking. Our findings provide information on the causal relationship between NC and LC, and highlight the objective differences in genetic variation among ethnicities.

## 1. Introduction

Lung cancer (LC), the most prevalent malignant tumor, poses a considerable threat to human health and imposes a considerable economic burden on the families of patients. Epidemiological studies have revealed that LC is associated not only with complex environmental factors such as smoking, outdoor air pollution and occupational exposure but also with genetic factors as independent risk factors 1. Research has demonstrated that individuals with a first-degree relative affected by cancer have a 2~4-times greater risk of developing LC than control individuals do (after adjustment for smoking-related factors) 2. Individuals exhibit varying susceptibilities to LC due to their distinct genetic backgrounds, despite having similar exposure intensities and durations 3. Early identification and prevention strategies for high-risk individuals in susceptible populations can enable timely intervention at the predisease or subclinical stage, thereby mitigating patient and familial distress and lessening the burden on society.

Neutrophils constitute approximately 60% of peripheral blood leukocytes, making them the most abundant type of innate immune cell 4. Neutrophils have various functions in host defense, including antimicrobial and antiviral activities, clearance of apoptotic cell debris, tissue repair and regeneration postinjury, and angiogenesis 5, 6. Conversely, neutrophils also contribute to disease pathogenesis via mechanisms such as tissue damage, chronic inflammation, and immune suppression via protease release. An important clinical indicator, the neutrophil count, reflects the state of the disease and can even predict its development. Numerous epidemiological studies and our previous research have indicated a strong correlation between neutrophil-related indicators in the circulation and the tumor microenvironment (TME) and the risk of LC 7-11. However, research has focused predominantly on association and mechanistic studies. There is little evidence of a causal relationship. Traditional observational epidemiological studies are unable to fully account for the intricate biological confounders and bidirectional causality between neutrophils and LC. In addition, the genetic characteristics of patients with lung cancer differ according to race. Hence, the genetic associations between neutrophil count and lung cancer across ethnicities warrant investigation. However, this clinically important topic has yet to be fully explored and discussed by scholars.

Therefore, we aimed to explore the genetic evidence of the causal relationship between neutrophil count and the risk of LC in East Asian (EAS) and European (EUR) populations via MR analysis.

## 2. Materials and Methods

### 2.1 Study design

This study employed a large-scale public GWAS summary database to perform two-sample bidirectional MR analyses between neutrophil counts (exposure) and lung cancer (outcome) risk to evaluate the causal effect in two different populations. Furthermore, a multivariable MR (MVMR) was conducted to assess the effects of confounding factors.

### 2.2 Data sources

The genetic instrumental variable (IV) data for the EAS population (ebi-a-GCST90018748) were sourced from a 2021 GWAS by Saori Sakaue *et al.*, which was published in *Nature Genetics;* the study involved 82,810 participants and yielded 12,491,034 SNPs 12. The LC data were obtained from a GWAS (ebi-a-GCST90018655) involving 178,726 individuals and 12,454,705 SNPs in the EAS population. The genetic variation data for neutrophils in the EUR population (ebi-a-GCST90002350) were derived from a 2020 meta-analysis by Ming-Huei Chen *et al.*, which was published in *Cell*, and involved 13,476 participants from transethnic populations, with a total of 33,797,434 SNPs 13. The LC data included 40,453 EUR individuals (7,877,791 SNPs), which were extracted from the large-scale GWAS data of the TRICL Consortium (ieu-a-985).

### 2.3 Data Analysis

#### 2.3.1 MR analysis

We extracted SNPs associated with the neutrophil count (NC) and removed IVs in linkage disequilibrium (LD) using filter conditions (r^2^=0.001, window size=10,000 kb) to control for bias. Information for each SNP was collected. SNPs with F statistics of <10 were considered weak instrumental variables and were excluded. The F statistic was calculated as:



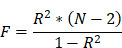



As the inverse-variance weighted (IVW) method has superior effectiveness and accuracy for data analysis compared with other methods, it was primarily used for the main analysis in this study on the basis of the satisfaction of three basic assumptions of MR analysis. Changes in LC risk caused by NC were expressed as odds ratios (ORs) and 95% confidence intervals (CIs). A value of *P* <0.05 was taken to indicate statistical significance.

#### 2.3.2 Sensitivity analysis

The pleiotropy test, heterogeneity test, and leave-one-out analysis were included in the sensitivity analysis.

We used the MR‒Egger intercept test and Mendelian randomization pleiotropy residual sum and outlier (MR-PRESSO) test to assess the impact of horizontal pleiotropy. If the intercept of the MR‒Egger test is not equal to 0 and the *P* value is <0.05, it indicates the objective presence of an intercept that cannot be ignored. A *P* value of <0.05 by the MR-PRESSO global test indicates the presence of outliers in the IVs. A *P* value of < 0.05 by Cochran's Q test indicates the presence of heterogeneity among IVs, and I^2^ values <25%, 25-50%, and >50% indicate low, moderate and high heterogeneity, respectively. The leave-one-out (LOO) method was used to calculate the MR results of the remaining IVs by repeatedly removing single SNPs from the IVs. The consistent effect estimates obtained upon the exclusion of all SNPs indicate the robustness and reliability of the results.

#### 2.3.3 Reverse causation test

To address the potential reverse causality, a reverse MR analysis was conducted. Specifically, IVs were extracted from the GWAS summary data of LC. The causal relationship between LC (exposure) and NC (outcome) was examined.

#### 2.3.4 Confounding factor analysis

Given the complexity of biological factors, we performed a multifactor MR analysis to eliminate potential confounding factors. Previous studies have established a clear causal link between smoking and both LC and the neutrophil count. Therefore, NC and smoking traits were considered exposure factors to assess their causal relationships with LC, with the aim of identifying factors that could impact effect estimation.

All analyses were conducted with R version 4.2.0. We used the TwoSampleMR package to conduct the MR analysis. The harmonize_data function was used for analysis of the consistency of the effect alleles of SNPs related to the outcome, after removing SNPs with palindromic structures to avoid the risk of errors introduced by chain issues. The steiger_filtering function was implemented to exclude SNPs with a greater impact on explaining the variance in the LC phenotype than that in the neutrophil count. The mr function was used for MR analysis. The mr_pleiotropy_test function was employed for the MR‒Egger test, whereas the run_mr_presso function was used for the MR-PRESSO test. The mr_heterogeneity function was used for heterogeneity testing. The mr_leaveneout function was employed for leave-one-out analysis.

## 3. Results

### 3.1 Characteristics of genetic instrumental variables

In the EAS population, a total of 37 SNPs associated with NC were identified, while 32 SNPs were identified in the EUR population. Tables S2 and S3 present information on the SNPs in the two populations. The F statistics for all IVs exceeded 10.

### 3.2 Association between NC and lung cancer according to MR analysis

**Figure 1:**
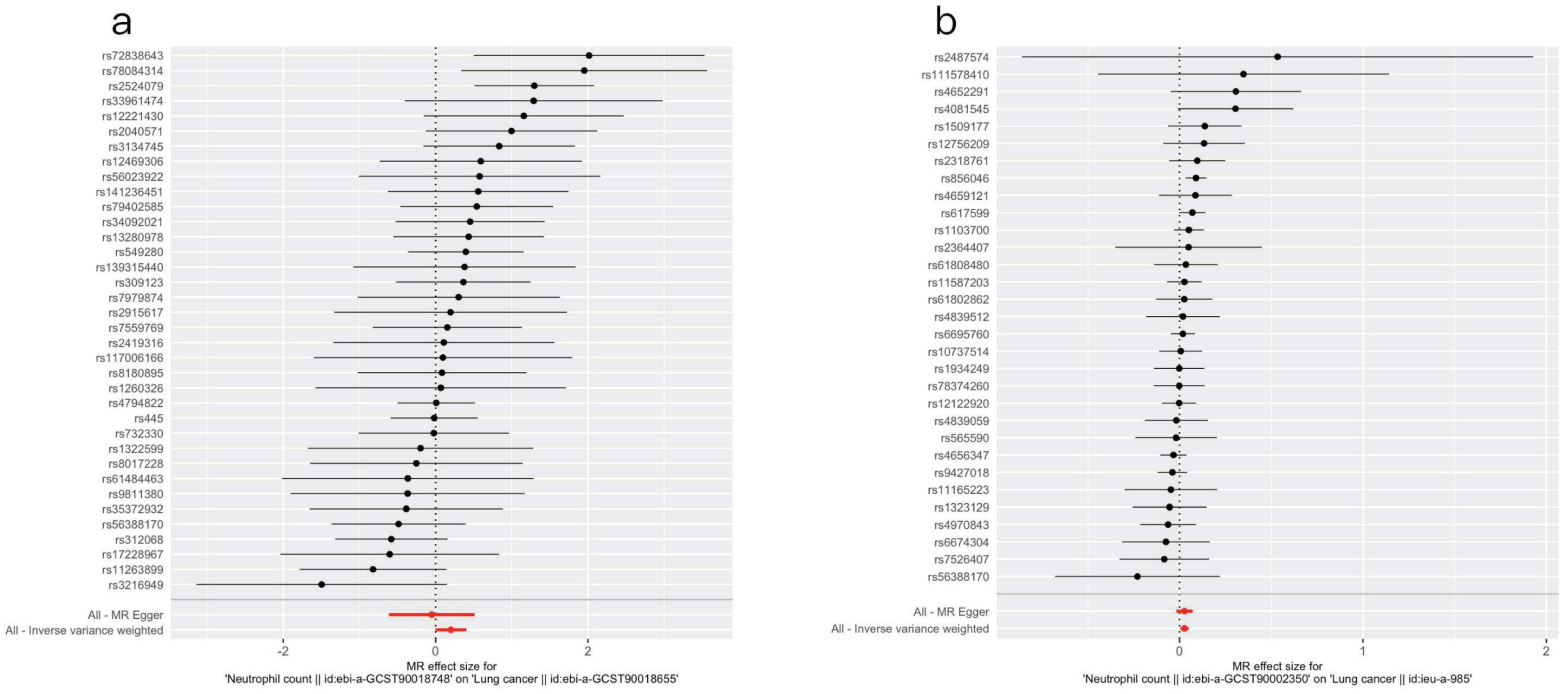
Forest plots of the association of neutrophil count with lung cancer (a) in East Asian populations and (b) in European populations.

After screening and matching, 36 SNPs were obtained from the EAS population, whereas 31 SNPs were obtained from the EUR population (Tables S2 and S3). Using the IVW, MR Egger, weighted median, simple mode, and weighted mode methods, the causal relationship between NC and LC was evaluated in both populations; the effect estimates are detailed in Table 1. The findings suggested no statistically significant causal association between genetically predicted NC and LC risk (*P*=0.052) in the EAS population. Consistent results were obtained using all the other analytical methods (*P* > 0.05). In the EUR population, a statistically significant causal association was observed between NC and LC (OR_IVW_=1.027, 95% CI: 1.005-1.050, *P* =0.017). Figure 1 visualizes the associations.

### 3.3 Sensitivity analysis

#### 3.3.1 Heterogeneity tests

Table 2 shows the heterogeneity results. In the EAS populations, the I^2^ value was approximately 25%, indicating a relatively low level of heterogeneity among the IVs. In the European populations, the I^2^ value was close to 0, indicating virtually no heterogeneity. Additionally, all the Cochran's Q p values were above 0.05, suggesting that heterogeneity could be disregarded. Figure S1 shows the funnel plots, which suggested that the scatter points were evenly distributed on both sides.

#### 3.3.2 Pleiotropy tests

The results of the pleiotropy tests are presented in Table 3. The MR‒Egger intercept P values were above 0.05 (*P*_EAS_=0.354, *P*_EUR_=0.991). The MR-PRESSO global test yielded similar findings (*P*_EAS_=0.09, *P*_EUR_=0.528).

#### 3.3.3 Leave-one-out sensitivity analysis

**Figure 2:**
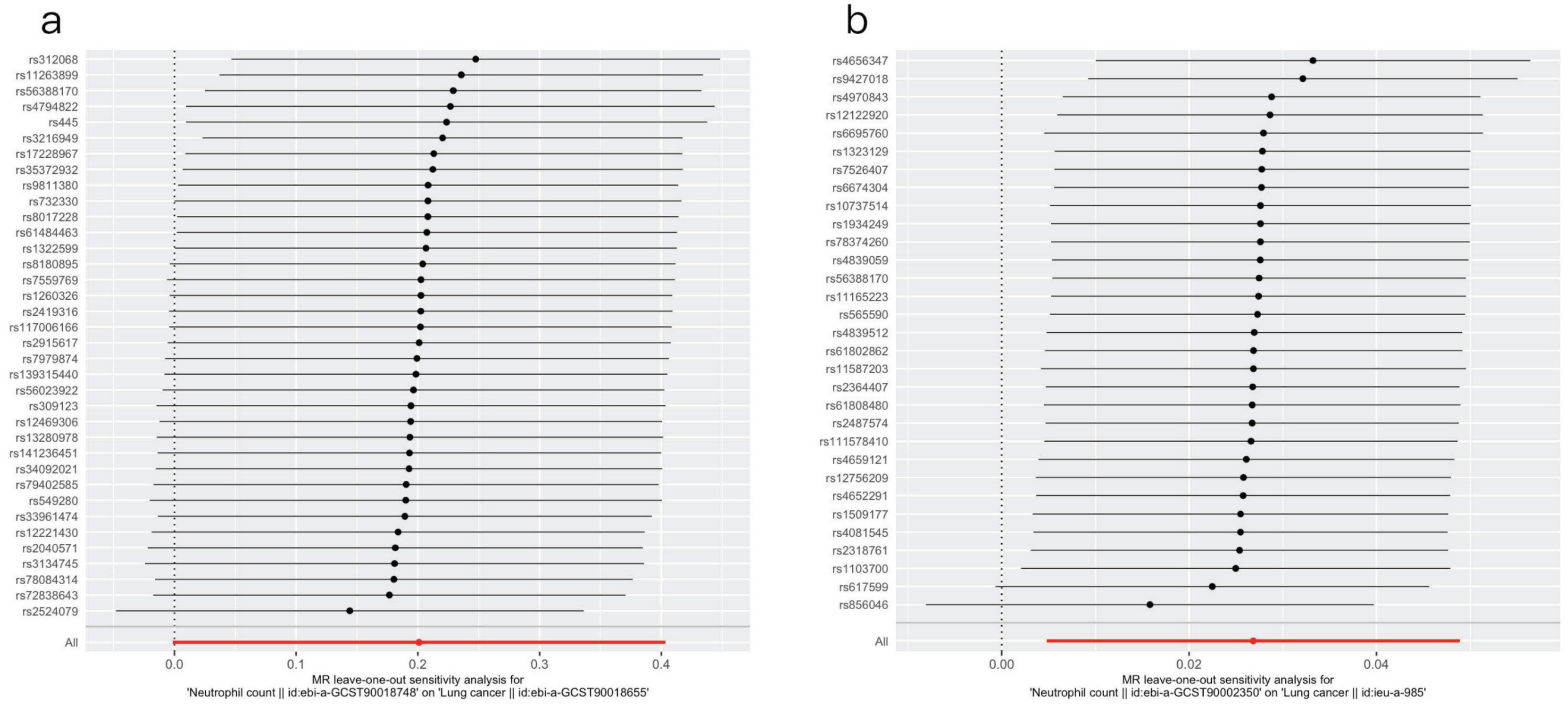
Leave-one-out plots for the causal effect of neutrophil count on lung cancer (a) in the East Asian population and (b) in the European population.

The results of the leave-one-out sensitivity test are displayed in Figure 2. The x-axis represents the effect estimates. The red line represents the fitted results. The overall fit did not substantially change after the SNPs were individually removed. These consistent results suggest that our findings are relatively robust and plausible.

### 3.4 Reverse Mendelian Randomization

Table 4 shows that there was no significant causal association between LC (exposure) and NC (outcome) (*P* > 0.05, in both populations), which suggested that the causal relationship between NC (exposure) and LC (outcome) in the EUR population was unidirectional.

### 3.5 Analyses of confounding factors

As shown in Table 5, after we adjusted for smoking, the significant causal relationship between NC and LC persisted (*P*_EUR_=0.042) in the EUR population. However, no association was detected in the EAS population.

## 4. Discussion

The findings revealed that an increased NC level was potentially linked to LC in EUR individuals, without interference from reverse causation. This causal association was absent in the EAS population. A multivariable analysis revealed that even after adjusting for smoking, a factor strongly causally related to LC, the significant causal relationship between NC and LC persisted.

Since the discovery of hematopoiesis-related genetic loci, MR analyses based on publicly available large GWAS databases have been performed. MR is an epidemiological method that uses instrumental variables to evaluate the causal relationship between exposure and outcomes. In 2016, a study published in *Cell* employed the MR method to investigate the causal mechanisms between blood cell indices and chronic diseases 14, and the findings suggested that the previously reported causal associations between WBC-related indicators and several chronic diseases may be due to confounding factors. The results of an observational and genetic study performed by a team from Copenhagen University Hospital revealed significant causal associations between NC and the risk of ischemic heart disease (OR=1.15, 95% CI: 1.08, 1.21), myocardial infarction (OR=1.22, 1.12, 1.34), and peripheral arterial disease (OR=1.19, 1.04, 1.36) 15. Neutrophils can drive tumor progression and metastasis through various pathways, including by promoting thrombosis and angiogenesis, remodeling the matrix, and impairing T-cell-dependent antitumor immunity 16, 17. Moreover, neutrophils bind to circulating tumor cells and enhance hematogenous metastasis by promoting tumor cell cycle progression 16, 17. The causal links between neutrophils and multiple cancer types have been studied. A study published in *BMC Medicine* in 2023 used the results of proteomic analysis of the plasma of 3,301 healthy individuals and publicly available GWAS summary data for multiple myeloma (MM) patients (598 cases and 180,756 controls) to investigate the causal relationship between 2,994 proteins and the MM risk 18. A total of 13 proteins were identified, with one neutrophil-related protein, NCF2, showing a positive correlation with the MM risk (OR=1.27, 95% CI: 1.12, 1.44). In 2021, Linda *et al.* reported a negative correlation between genetic prediction of the neutrophil-to-lymphocyte ratio and susceptibility to childhood acute lymphoblastic leukemia (ALL) (OR=0.67, *P*=3.1E^-4^) on the basis of GWAS databases 19. Scholars from Qilu Hospital of Shandong University (China) employed three GWAS databases—INTERVAL, UKB, and the UK BiLEVE—to infer the causal relationships between the numbers of eight phenotypes of WBCs and the risk of hepatocellular carcinoma (HCC)^20^. The authors reported a negative correlation between the total number of alkaline neutrophils and the HCC risk (OR=0.437, 95% CI: 0.252, 0.757).

Lung cancer is a multifactorial disease influenced by both genetic and environmental factors. Early detection of susceptibility loci for LC and prompt intervention with targeted treatments can significantly influence the development and prognosis of this disease. An epidemiological longitudinal study and MR analysis was published in *Nature* in 2022 21. The findings revealed that lineage-specific genetic variations can impact susceptibility to CHIP, which is significantly causally linked to nonmelanoma skin cancer, melanoma, and lung cancer. Ying Zhu *et al.* reported a causal relationship between an elevated platelet count and an increased risk of LC, with an overall 62% increase in the risk of non-small cell lung cancer (OR=1.62, 95% CI: 1.15, 2.27) and a 200% increase in the risk of small cell lung cancer (OR=3.00, 95% CI: 1.27, 7.06) 22. A study by the National Clinical Research Center for Respiratory Diseases (China) analyzed GWAS data from EUR and EAS populations, and revealed that an increase in eosinophil count was causally associated with an increased risk of LC in EAS populations, particularly for lung squamous cell carcinoma (OR=1.28, 95% CI: 1.04, 1.57) 23. In a recent study by Chinese scholars, genetically driven increases in reticulocyte count (OR=0.923, 95% *CI*: 0.872, 0.976) and hematocrit (OR=0.845, 95% CI: 0.783, 0.913) were found to be causally associated with a decreased LC risk 24.

While many epidemiological studies support the close association of neutrophils with LC in the circulation and tumor microenvironment, there is insufficient evidence of a causal link. Yang *et al.* investigated the causal relationships between 23 hematological traits and LC risk 24. However, in this study there was no significant genetic causal relationship between neutrophils and LC or its three subtypes. Although the literature on the association between neutrophils and LC is limited, reports on the associations between neutrophils and lung diseases or traits could provide information. During systemic infections, neutrophils are typically sequestered in the lung and liver, which may allow bacteria to evade the innate immune response or permit neutrophils to form neutrophil extracellular traps (NETs), which capture and kill bacteria in the bloodstream 6, 17. Neutrophils with pathogen-killing abilities significantly regulate the functions of other immune cells and their recruitment to the lung. They play a crucial role in innate immunity during lung inflammation. Recent studies have highlighted a causal relationship between neutrophils and lung diseases or related traits. An observational study published in 2021 demonstrated an independent correlation between total WBC count, neutrophil count, and lung function (*P* < 0.05) 25. MR analysis revealed that genetic prediction of NC was associated with reduced lung function parameters, FVC (*P* = 0.021) and FEV1 (*P* = 0.043). Zhifa Han *et al.* conducted a bidirectional MR analysis of blood cell indices and lung function and reported that COPD exacerbation or a decrease in FEV1/FVC could lead to an increased NC level, with odds ratios of 1.03 (95% *CI:* 1.01, 1.05) and 0.947 (95% CI: 0.91, 0.986), respectively 26. This emphasized the importance of considering the direction of the causality between exposure and disease. The conflicting results of prior studies on the causal relationship between neutrophils and diseases suggest the need for further investigation.

This study adjusted for smoking, which is a confounding factor closely associated with LC. Despite the strong association between smoking and LC in both populations, MVMR analysis indicated a significant causal link between NC and LC in EUR individuals, confirming the stability of the results. Nimesh A Jayasuriya *et al.* investigated the association between smoking and blood cell count among 11,083 individuals from the general suburban population of Denmark in the GESUS study 27. Moreover, causal relationships among smoking, blood cell counts, and myeloproliferative neoplasms (MPNs) were examined in a total of 2,307,745 participants. The results revealed that tobacco consumption among current smokers was associated with increases in WBCs, neutrophils, and other indicators. Subsequent observational analyses revealed a dose‒response relationship with smoking history, tobacco intake, cell counts/indices, and MPN risk compared with nonsmokers. This may be attributable to changes in blood cell proportions, leading to an increased risk of thrombosis and subsequently an elevated risk of secondary cancers among smokers. These studies suggest that increased blood cell counts in smokers may indicate a cancer risk.

This study has several strengths. Causal inference between exposure and outcome was conducted in two ethnicities, although there was a significant association only in the European population. This underscores the substantial genetic variances among ethnicities, suggesting the need for cautious generalization and interpretation of the results of genetic studies. Several methodologies, such as the MR‒Egger test, MR-PRESSO test, and LOO analysis, were employed to ensure the stability of the results and the rigor of the conclusions. We also acknowledge that there are several limitations to this study. First, despite conducting a variety of tests to ensure the robustness of the results, the errors cannot be rules out. Research has suggested that nearly half of MR studies are susceptible to varying degrees of genetic pleiotropy and that the existence of unknown pathway deviations can impact stability 28. In addition, given that MR relies on a genetic perspective and infers causal relationships via instrumental variable methods, particular caution is needed when interpreting the results, which should consider real-world factors. Furthermore, as data from individual sources were not obtained, stratified analyses based on factors such as age and sex were not performed. Finally, we were unable to access the major clinical parameters of the dataset used in this study. Conducting statistical comparisons of important clinical indicators across different ethnic groups is crucial, as it could provide valuable information for clinical applications. In future, we will incorporate accessible clinical characteristics into our research platform to assess the generalizability of our findings across various clinical scenarios.

## 5. Conclusion

The exploration of causal relationships is important for disease prevention policies and etiological mechanism studies. Our study provides information on the causal relationship between neutrophils and LC, and the findings emphasize the objective differences in genetic variations among ethnicities. This study revealed a causal relationship between increased NC levels and LC risk in the EUR population. This urges us to consider the potential endogenous and exogenous effects of blood cell indices on organisms, offers a theoretical basis for identifying biological markers and therapeutic targets for LC, and will enable the formulation of scientifically guided strategies for its early detection, diagnosis, and treatment. Further research should comprise deeper and larger-scale systematic studies, the results of which will supplement these findings.

## Supplementary Material

Supplementary figure and tables.

## Figures and Tables

**Table 1:** Results of MR analysis of neutrophil counts in patients with lung cancer.

Method	EAS Population					EUR Population				
	nsnp	β	SE	OR (95% CI)	P	nsnp	β	SE	OR (95% CI)	P
**IVW**	**36**	**0.201**	**0.103**	**1.223 (0.999, 1.497)**	**0.052**	**31**	**0.027**	**0.011**	**1.027 (1.005, 1.050)**	**0.017**
MR Egger	36	-0.050	0.287	0.951 (0.542, 1.668)	0.862	31	0.027	0.023	1.027 (0.982, 1.075)	0.249
Weighted median	36	0.085	0.142	1.089 (0.825, 1.438)	0.547	31	0.018	0.018	1.019 (0.984, 1.054)	0.293
Simple mode	36	0.225	0.262	1.252 (0.750, 2.091)	0.397	31	0.003	0.028	1.003 (0.950, 1.060)	0.905
Weighted mode	36	0.116	0.202	1.123 (0.756, 1.668)	0.569	31	0.017	0.024	1.017 (0.971, 1.066)	0.482

EAS, East Asian; EUR, European; SE, standard error; IVW, inverse-variance weighted; OR, odds ratio; CI, confidence interval Bold typeface shows the IVW method analysis result, which is the main result used in this study.

**Table 2:** Results of the heterogeneity tests.

Population	Method	Q	Cochran's Q pval	I^2^
EAS	IVW	47.067	0.084	0.256
EUR	IVW	27.724	0.585	0

EAS, East Asian; EUR, European; IVW, inverse-variance weighted

**Table 3:** Results of the pleiotropy tests.

Population	Method	Intercept	SE	*P*
EAS	MR‒Egger intercept test	0.013	0.014	0.354
	MR-PRESSO global test	-	-	0.090
EUR	MR‒Egger intercept test	-7.65E-05	0.007	0.991
	MR-PRESSO global test	-	-	0.528

EAS, East Asian; EUR, European; SE, standard error.

**Table 4:** Reverse MR analysis results of lung cancer samples with different neutrophil counts.

Method	EAS Population					EUR Population			
	nsnp	β	SE	*P*		nsnp	β	SE	*P*
IVW	7	0.021	0.016	0.187		10	0.057	0.036	0.113
MR Egger	7	-0.045	0.595	0.943		10	0.053	0.082	0.538
Weighted median	7	0.002	0.017	0.921		10	0.063	0.042	0.141
Simple mode	7	0.001	0.024	0.983		10	0.038	0.078	0.633
Weighted mode	7	0.001	0.022	0.982		10	0.050	0.044	0.281

EAS, East Asian; EUR, European; IVW, inverse-variance weighted; SE, standard error

**Table 5:** Results of the confounding factor analyses.

Population	GWAS ID	Confounding Factor	nsnp	β	SE	OR (95% CI)	*P*
EAS	ebi-a-GCST90018748	Neutrophil count	31	0.174	0.120	1.190 (0.941, 1.504)	0.147
	ieu-b-5071	Cigarettes per day	5	1.001	0.201	2.720 (1.833, 4.037)	6.81E-07
EUR	ebi-a-GCST90002350	Neutrophil count	28	0.043	0.021	1.044 (1.002, 1.088)	0.042
	ieu-b-25	Cigarettes per day	19	1.076	0.11	2.933 (2.365, 3.639)	1.224E-22

EAS, East Asian; EUR, European; SE, standard error; OR, odds ratio; CI, confidence interval

## Abbreviations

MR: Mendelian randomization
MVMR: Multivariable mendelian randomization
SNPs: Single nucleotide polymorphisms
NC: Neutrophil count
LC: Lung cancer
IV: Instrumental variable
IVW: Inverse-variance weighted
MR-PRESSO: Mendelian randomization pleiotropy residual sum and outlier
LD: Linkage disequilibrium
GWAS: Genome-wide association studies
EUR: European
EAS: East Asian
EA: Effect allele
OA: Other allele
EAF: Effect allele frequency
OR: Odds ratio
SE: standard error
CI: Confidence interval
WBC: White blood cell

## References

[1] Swanton C, Govindan R (2016). Clinical Implications of Genomic Discoveries in Lung Cancer. N Engl J Med.

[2] Cannon-Albright LA, Carr SR, Akerley W (2019). Population-Based Relative Risks for Lung Cancer Based on Complete Family History of Lung Cancer. J Thorac Oncol.

[3] Vachani A, Sequist LV, Spira A (2017). AJRCCM: 100-Year Anniversary. The Shifting Landscape for Lung Cancer: Past, Present, and Future. Am J Respir Crit Care Med.

[4] Liew PX, Kubes P (2019). The Neutrophil's Role During Health and Disease. Physiol Rev.

[5] Coffelt SB, Wellenstein MD, de Visser KE (2016). Neutrophils in cancer: neutral no more. Nat Rev Cancer.

[6] Kolaczkowska E, Kubes P (2013). Neutrophil recruitment and function in health and inflammation. Nat Rev Immunol.

[7] Borné Y, Smith JG, Nilsson PM, Melander O, Hedblad B, Engström G (2016). Total and Differential Leukocyte Counts in Relation to Incidence of Diabetes Mellitus: A Prospective Population-Based Cohort Study. PLoS One.

[8] Shiels MS, Pfeiffer RM, Hildesheim A, Engels EA, Kemp TJ, Park JH (2013). Circulating inflammation markers and prospective risk for lung cancer. J Natl Cancer Inst.

[9] Nøst TH, Alcala K, Urbarova I, Byrne KS, Guida F, Sandanger TM (2021). Systemic inflammation markers and cancer incidence in the UK Biobank. Eur J Epidemiol.

[10] Wang F, Chen L, Wang Z, Xu Q, Huang H, Wang H (2022). Prognostic value of the modified systemic inflammation score in non-small-cell lung cancer with brain metastasis. Cancer Cell Int.

[11] Liu W, Ren S, Yang L, Xiao Y, Zeng C, Chen C (2023). The predictive role of hematologic markers in resectable nsclc patients treated with neoadjuvant chemoimmunotherapy: a retrospective cohort study. Int J Surg.

[12] Sakaue S, Kanai M, Tanigawa Y, Karjalainen J, Kurki M, Koshiba S (2021). A cross-population atlas of genetic associations for 220 human phenotypes. Nat Genet.

[13] Chen MH, Raffield LM, Mousas A, Sakaue S, Huffman JE, Moscati A (2020). Trans-ethnic and Ancestry-Specific Blood-Cell Genetics in 746,667 Individuals from 5 Global Populations. Cell.

[14] Astle WJ, Elding H, Jiang T, Allen D, Ruklisa D, Mann AL (2016). The Allelic Landscape of Human Blood Cell Trait Variation and Links to Common Complex Disease. Cell.

[15] Luo J, Thomassen JQ, Nordestgaard BG, Tybjærg-Hansen A, Frikke-Schmidt R (2023). Neutrophil counts and cardiovascular disease. Eur Heart J.

[16] Hedrick CC, Malanchi I (2022). Neutrophils in cancer: heterogeneous and multifaceted. Nat Rev Immunol.

[17] Mantovani A, Cassatella MA, Costantini C, Jaillon S (2011). Neutrophils in the activation and regulation of innate and adaptive immunity. Nat Rev Immunol.

[18] Wang Q, Shi Q, Wang Z, Lu J, Hou J (2023). Integrating plasma proteomes with genome-wide association data for causal protein identification in multiple myeloma. BMC Med.

[19] Kachuri L, Jeon S, DeWan AT, Metayer C, Ma X, Witte JS (2021). Genetic determinants of blood-cell traits influence susceptibility to childhood acute lymphoblastic leukemia. Am J Hum Genet.

[20] Pan GQ, Yang CC, Shang XL, Dong ZR, Li T (2022). The causal relationship between white blood cell counts and hepatocellular carcinoma: a Mendelian randomization study. Eur J Med Res.

[21] Kessler MD, Damask A, O'Keeffe S, Banerjee N, Li D, Watanabe K (2022). Common and rare variant associations with clonal haematopoiesis phenotypes. Nature.

[22] Zhu Y, Wei Y, Zhang R, Dong X, Shen S, Zhao Y (2019). Elevated Platelet Count Appears to Be Causally Associated with Increased Risk of Lung Cancer: A Mendelian Randomization Analysis. Cancer Epidemiol Biomarkers Prev.

[23] Wang Z, Chen B, Fu Y, Ou C, Rong Q, Kong X (2022). Eosinophilia and Lung Cancer: Analysis From Real-World Data and Mendelian Randomization Study. Front Med (Lausanne).

[24] Yang Z, He H, He G, Zeng C, Hu Q (2024). Investigating Causal Effects of Hematologic Traits on Lung Cancer: A Mendelian Randomization Study. Cancer Epidemiol Biomarkers Prev.

[25] Wu X, Wang C, Li H, Meng H, Jie J, Fu M (2021). Circulating white blood cells and lung function impairment: the observational studies and Mendelian randomization analysis. Ann Med.

[26] Han Z, Hu H, Yang P, Li B, Liu G, Pang J (2022). White blood cell count and chronic obstructive pulmonary disease: A Mendelian Randomization study. Comput Biol Med.

[27] Jayasuriya NA, Kjaergaard AD, Pedersen KM, Sørensen AL, Bak M, Larsen MK (2020). Smoking, blood cells and myeloproliferative neoplasms: meta-analysis and Mendelian randomization of 2·3 million people. Br J Haematol.

[28] Verbanck M, Chen CY, Neale B, Do R (2018). Detection of widespread horizontal pleiotropy in causal relationships inferred from Mendelian randomization between complex traits and diseases. Nat Genet.

