# Supplementary material for: The causal association between neutrophil counts and the risk of lung cancer: a Mendelian randomization study
Source: J Cancer. 2025 Jan 1;16(1):331–8. doi: 10.7150/jca.100884 (PMC11660127; doi:10.7150/jca.100884)
Supplement: Supplementary file 1 — Supplementary figure and tables. [file jcav16p0331s1.pdf]

Supplements

Table S1. Characteristics of datasets.

GWAS ID	Year	Population	ncase	ncontrol	Traits	Sample size	nSNPs
ebi-a-GCST90018748	2021	EAS	NA	NA	Neutrophil count	82,810	12,491,034
ebi-a-GCST90002350	2020	Mix	NA	NA	Neutrophil count	13,476	33,797,434
ebi-a-GCST90018655	2021	EAS	174,282	4,444	Lung Cancer	178,726	12,454,705
ieu-a-987	NA	EUR	55,586	29,863	Lung cancer	85,449	10,439,018
ieu-b-5071	2019	EAS	NA	NA	Cigarettes per day	72,655	6,108,828
ieu-b-25	2019	EUR	NA	NA	Cigarettes per day	337,334	11,913,712

EAS, East Asian; EUR, European; nSNPs, number of SNPs; NA, not applicable.

Table S2. Characteristics of genetic instrumental variables in the **East Asian** population.

SNP	chr	pos	EA	OA	EAF	*β*	SE	*P*	F-statistic	GENCODE genes	Variation Type	Functional annotation
rs11263899	1	36915345	T	C	0.395	0.045	0.005	9.70E-22	91.785	OSCP1<br>LOC107984940:500B	SNV	Intron Variant (OSCP1)<br>Downstream Variant (LOC107984940:500B)
rs1260326	2	27730940	C	T	0.440	-0.026	0.005	2.20E-08	31.339	GCKR	SNV	Missense Variant
rs7559769	2	136785353	T	C	0.280	0.049	0.005	4.10E-21	88.916	11kb 3' of AC093391.2	SNV	None
rs309123	2	136761175	G	C	0.288	0.054	0.005	1.50E-25	109.125	DARS1-AS1	SNV	Intron Variant
rs79402585	2	219106172	A	G	0.095	-0.076	0.008	5.60E-20	83.769	ARPC2	SNV	Intron Variant
rs61484463	2	111929881	C	T	0.174	0.034	0.006	2.30E-08	31.246	5.3kb 3' of BCL2L11	SNV	None
rs12469306	2	160705485	G	A	0.823	-0.042	0.006	4.40E-12	47.938	LY75<br>LY75-CD302	SNV	Intron Variant
rs17228967	2	219248396	C	T	0.047	-0.070	0.011	1.50E-10	41.071	SLC11A1	SNV	Intron Variant
rs9811380	3	128321226	G	A	0.147	-0.040	0.007	2.00E-09	35.977	18kb 3' of RPN1	SNV	None
rs549280	4	74971196	A	G	0.560	-0.057	0.005	6.50E-34	147.382	6.2kb 5' of CXCL2	SNV	None
rs56023922	4	87916086	C	A	0.330	0.029	0.005	6.10E-09	33.790	AFF1	SNV	Intron Variant
rs33961474	4	75126735	A	AT	0.241	0.030	0.005	4.80E-08	29.812	MTHFD2L	SNV	Intron Variant
rs7725218*	5	1282414	A	G	0.352	0.041	0.005	9.80E-16	64.462	TERT	SNV	Intron Variant
rs35372932	6	32564985	T	C	0.221	-0.041	0.006	4.90E-13	52.236	7.4kb 5' of HLA-DRB1	SNV	None
rs1322599	6	16758425	T	C	0.625	-0.030	0.005	4.40E-10	38.940	ATXN1	SNV	Intron Variant
rs72838643	6	29843390	G	A	0.373	0.029	0.005	1.20E-09	36.987	HCP5B<br>LOC124905390<br>LOC124905394	SNV	Upstream Variant (HCP5B:2KB)<br>Non Coding Transcript Variant (LOC124905390)<br>Non Coding Transcript Variant (LOC124905394)
rs2524079	6	31242174	A	G	0.272	0.061	0.005	6.30E-32	138.274	2.3kb 5' of HLA-C	SNV	None
rs3134745	6	31242762	T	C	0.093	0.074	0.008	1.40E-20	86.443	2.9kb 5' of HLA-C	SNV	None
rs78084314	6	33863667	T	C	0.080	0.049	0.009	9.40E-09	32.959	LINC01016<br>LOC105375026	SNV	Intron Variant (LINC01016)<br>Non Coding Transcript Variant (LOC105375026)
rs56388170	7	28724374	T	G	0.213	0.061	0.006	2.00E-25	108.590	CREB5	SNV	Intron Variant
rs445	7	92408370	T	C	0.314	-0.080	0.005	2.40E-58	259.366	CDK6	SNV	Intron Variant
rs732330	7	92203716	C	T	0.369	0.047	0.005	8.30E-21	87.525	FAM133B	SNV	Intron Variant
rs2915617	8	30272310	G	C	0.860	0.042	0.007	1.80E-09	36.189	RBPMS	SNV	Intron Variant (RBPMS)<br>Upstream Variant (LOC112268025:2KB)
rs8180895	8	130506352	A	G	0.133	-0.060	0.007	6.40E-17	69.860	CCDC26	SNV	Intron Variant
rs13280978	8	61704055	T	C	0.776	-0.052	0.006	1.10E-20	86.982	CHD7	SNV	Intron Variant

rs34092021	8	130637901	A	G	0.231	-0.052	0.006	4.30E-21	88.846	CCDC26	SNV	Intron Variant
rs12221430	10	99061386	G	C	0.659	0.034	0.005	2.10E-12	49.425	9kb 5' of ARHGAP19	SNV	None
rs139315440	10	111946207	A	G	0.341	0.031	0.005	2.50E-10	40.043	21kb 5' of MXI1	SNV	None
rs2419316	10	112032958	G	A	0.388	-0.031	0.005	2.10E-10	40.337	MXI1	SNV	Intron Variant
rs7979874	12	110087038	T	C	0.144	0.050	0.007	4.70E-12	47.822	14kb 3' of 7SK	SNV	None
rs2040571	12	111357727	A	G	0.217	0.047	0.006	3.70E-16	66.402	MYL2	SNV	Intron Variant
rs8017228	14	25449226	C	A	0.475	0.031	0.005	4.00E-11	43.597	STXBP6	SNV	Intron Variant
rs4794822	17	38156712	T	C	0.517	0.084	0.005	1.30E-73	329.382	2.5kb 3' of PSMD3	SNV	None
rs141236451	18	42085693	CA	C	0.241	0.042	0.005	9.10E-15	60.085	LINC01478	Indel	Intron Variant
rs117006166	18	21893930	A	G	0.176	-0.035	0.006	4.50E-08	29.907	OSBPL1A	SNV	Intron Variant
rs312068	19	3207338	C	T	0.938	0.137	0.011	3.00E-36	158.028	NCLN	SNV	Intron Variant
rs3216949	19	993730	GC	G	0.671	0.028	0.005	2.30E-08	31.246	WDR18	Indel	Intron Variant

EA, effect allele; OA, other allele; EAF, Effect allele frequency; SE, standard error; Indel, Insertion and Deletion; SNV, Single Nucleotide Variation.
The information of GENCODE genes, Variation Type and Functional annotation are from the website of HaploReg v4.2 (https://pubs.broadinstitute.org/mammals/haploreg/haploreg.php) or dbSNP (https://www.ncbi.nlm.nih.gov/snp/).
*The MR-PRESSO test indicated that it was an outlier, which was removed in the subsequent analysis.

Table S3. Characteristics of genetic instrumental variables in the **European** population.

SNP	chr	pos	EA	OA	EAF	*β*	SE	*P*	F-statistic	GENCODE genes	Variation Type	Functional annotation
rs9427018	1	159382290	T	C	0.763	-0.415	0.017	6.75E-130	588.282	OR10J1	SNV	Intron Variant
rs617599	1	160162959	A	G	0.091	0.411	0.024	6.14E-68	303.569	CASQ1	SNV	Intron Variant
rs7526407	1	147070609	C	T	0.230	0.178	0.016	1.29E-27	118.727	BCL9	SNV	Intron Variant
rs4659121	1	119320954	G	T	0.133	0.146	0.020	1.80E-13	54.287	105kb 3' of TBX15	SNV	None
rs2487574	1	120395902	T	C	0.492	-0.080	0.013	2.14E-10	40.392	40kb 3' of ADAM30	SNV	None
rs4081545	1	173159516	C	T	0.747	-0.096	0.016	1.25E-09	36.942	TNFSF4	SNV	Intron Variant
rs4656347	1	161996062	C	T	0.145	0.454	0.021	4.34E-108	488.039	2.4kb 5' of OLFML2B	SNV	None
rs6674304	1	116887742	C	T	0.764	-0.261	0.019	3.42E-44	194.631	28kb 5' of ATP1A1	SNV	None
rs4839059	1	116792835	G	A	0.175	0.191	0.019	2.49E-24	103.728	28kb 5' of U3	SNV	None
rs4970843	1	109887191	C	T	0.104	0.192	0.023	1.94E-16	67.762	SORT1	SNV	Intron Variant
rs4652291	1	178028711	T	C	0.747	-0.113	0.017	2.02E-11	45.019	22kb 5' of SEC16B	SNV	None
rs12738149*	1	105487899	T	C	0.045	0.233	0.039	1.60E-09	36.461	644kb 3' of RP11-251P6.1	SNV	None
rs11165223	1	95174500	A	G	0.113	0.124	0.023	4.53E-08	29.950	SLC44A3-AS1	SNV	Intron Variant
rs6695760	1	161885545	C	G	0.130	0.467	0.021	4.36E-106	478.859	ATF6	SNV	Intron Variant
rs12122920	1	151588735	A	G	0.112	0.318	0.023	6.94E-44	193.214	SNX27	SNV	Intron Variant
rs2318761	1	150257714	G	A	0.808	-0.194	0.019	7.25E-25	106.167	CIART	SNV	Intron Variant
rs2364407	1	155970120	C	T	0.512	0.119	0.013	6.47E-21	88.148	ARHGEF2-AS1	SNV	Intron Variant
rs78374260	1	164561086	T	C	0.015	0.376	0.055	5.92E-12	47.429	PBX1	SNV	Intron Variant
rs1323129	1	175595997	C	T	0.850	-0.153	0.025	8.86E-10	37.618	TNR	SNV	Intron Variant
rs61808480	1	120048422	A	T	0.016	0.345	0.061	1.37E-08	32.272	1.4kb 5' of HSD3B1	SNV	Upstream Variant
rs565590	1	100846956	C	T	0.848	-0.133	0.024	2.04E-08	31.504	CDC14A	SNV	Intron Variant
rs856046	1	158987941	G	A	0.158	0.644	0.020	1.00E-200	1064.118	IFI16	SNV	Intron Variant
rs1103700	1	158210602	C	T	0.193	0.444	0.018	2.61E-133	603.987	13kb 5' of CD1A	SNV	None
rs111578410	1	158355928	C	T	0.416	-0.173	0.013	2.37E-43	190.763	12kb 3' of OR10T2	SNV	None
rs1509177	1	95999282	A	G	0.115	0.145	0.022	8.92E-11	42.108	RP11-286B14.1	SNV	None
rs10737514	1	165497144	T	C	0.126	0.253	0.022	1.27E-31	137.047	LRRC52-AS1	SNV	Intron Variant
rs4839512	1	116569214	T	C	0.217	0.188	0.017	5.93E-30	129.425	SLC22A15	SNV	Intron Variant
rs11587203	1	163147481	T	C	0.031	0.425	0.041	1.24E-25	109.671	RGS5<br>RGS5-AS1	SNV	Intron Variant
rs1934249	1	118049831	T	A	0.056	0.247	0.029	4.58E-17	70.610	MAN1A2	SNV	Intron Variant
rs61802862	1	165759199	C	A	0.043	0.249	0.036	4.09E-12	48.151	TMCO1	SNV	None
rs12756209	1	103399207	C	A	0.136	0.141	0.020	4.75E-12	47.860	COL11A1	SNV	Intron Variant
rs56388170	7	28724374	T	G	0.534	0.074	0.013	9.15E-09	33.064	CREB5	SNV	Intron Variant

EA, effect allele; OA, other allele; EAF, Effect allele frequency; SE, standard error; SNV, Single Nucleotide Variation.
The information of GENCODE genes, Variation Type and Functional annotation are from the website of HaploReg v4.2 (https://pubs.broadinstitute.org/mammals/haploreg/haploreg.php) or dbSNP (https://www.ncbi.nlm.nih.gov/snp/).

*This SNP was not successfully matched in MR Analysis.

Table S4. The specific frequency comparison of rs56388170 between the two populations.

Population	EA	OA	EAF	Sample size*	Frequency of EA[a]	Frequency of OA[b]	*P*[+]
EAS	T	G	0.213	82810	17639	65171	<0.001
EUR	T	G	0.534	13476	7196	6280	

EA, effect allele; OA, other allele; EAF, Effect allele frequency; EAS, East Asian; EUR, European.

*The data of Sample size are from Table S1.

[a] EAF multiplied by by the number of sample size equals frequency of EA.

[b] The number of sample size minus frequency of EA is frequency of OA.

[+]The statistical method used is Chi-square test.

Table S5. The frequency difference comparison of 67 SNPs between the European and East Asian populations.

				Europe		East Asian		
SNP	Study	Ref Allele	Alt Allele	Sample Size	Alt Allele Fre	Sample Size	Alt Allele Fre	*P*+
rs9427018	1000Genomes_30x	C	T	1266	0.3444	1170	0.6949	<0.001
rs617599	1000Genomes_30x	G	A	1266	0.3681	1170	0.5496	<0.001
rs7526407	1000Genomes_30x	C	T	1266	0.1169	1170	0.3803	<0.001
rs4659121	1000Genomes_30x	T	G	1266	0.4913	1170	0.0906	<0.001
rs2487574	1000Genomes_30x	C	T	1266	0.0158	1170	0.0752	<0.001
rs4081545	1000Genomes_30x	T	C	1266	0.3199	1170	0.1077	<0.001
rs4656347	1000Genomes_30x	T	C	1266	0.6864	1170	0.4274	<0.001
rs6674304	1000Genomes_30x	T	C	1266	0.0671	1170	0.0671	**0.517**
rs4839059	1000Genomes_30x	A	G	1266	0.7401	1170	0.7214	**0.159**
rs4970843	1000Genomes_30x	T	C	1266	0.4842	1170	0.6103	<0.001
rs4652291	1000Genomes_30x	C	T	1266	0.1611	1170	0.2145	<0.001
rs12738149	1000Genomes_30x	C	T	1266	0.2085	1170	0.0077	<0.001
rs11165223	1000Genomes_30x	G	A	1266	0.4384	1170	0.2876	<0.001
rs6695760	1000Genomes_30x	G	C	1266	0.7014	1170	0.2932	<0.001
rs12122920	1000Genomes_30x	G	A	1266	0.5592	1170	0.1923	<0.001
rs2318761	1000Genomes_30x	A	G	1266	0.5221	1170	0.8197	<0.001
rs2364407	1000Genomes	T	C	1006	0.8648	1008	0.9643	<0.001
rs78374260	1000Genomes_30x	C	T	1266	0.0885	1170	0	<0.001
rs1323129	1000Genomes_30x	T	C	1266	0.5845	1266	0.5701	**0.247**
rs61808480	1000Genomes_30x	T	A	1266	0.0987	1266	0.0009	<0.001
rs565590	1000Genomes_30x	T	C	1266	0.5814	1266	0.6632	<0.001
rs856046	1000Genomes_30x	A	G	1266	0.8057	1266	0.5034	<0.001
rs1103700	1000Genomes_30x	T	C	1266	0.8073	1266	0.5103	<0.001
rs111578410	1000Genomes_30x	T	C	1266	0.015	1266	0	<0.001
rs1509177	1000Genomes_30x	G	A	1266	0.5506	1266	0.4821	<0.001
rs10737514	1000Genomes_30x	C	T	1266	0.5458	1266	0.7085	<0.001
rs4839512	1000Genomes_30x	C	T	1266	0.8144	1266	0.7496	<0.001
rs11587203	1000Genomes_30x	C	T	1266	0.1524	1266	0.0051	<0.001
rs1934249	1000Genomes_30x	A	T	1266	0.2385	1266	0.3735	<0.001
rs61802862	1000Genomes_30x	A	C	1266	0.1943	1266	0.0009	<0.001
rs12756209	1000Genomes_30x	A	C	1266	0.6145	1266	0.7094	<0.001
rs11263899	1000Genomes_30x	T	C	1266	0.3128	1170	0.3051	**0.358**
rs1260326	1000Genomes_30x	C	T	1266	0.5861	1170	0.5188	<0.001
rs7559769	1000Genomes_30x	T	C	1266	0.0134	1170	0.2709	<0.001
rs309123	1000Genomes_30x	G	C	1266	0.203	1170	0.3846	<0.001
rs79402585	1000Genomes_30x	A	G	1266	0.0166	1170	0.1077	<0.001
rs61484463	1000Genomes_30x	C	T	1266	0.0632	1170	0.1077	<0.001
rs12469306	1000Genomes_30x	G	A	1266	0.8649	1170	0.7632	<0.001

rs17228967	1000Genomes_30x	C	T	1266	0.0616	1170	0.0385	0.006
rs9811380	1000Genomes_30x	G	A	1266	0.2575	1170	0.235	**0.108**
rs549280	1000Genomes_30x	A	G	1266	0.6453	1170	0.4735	<0.001
rs56023922	1000Genomes_30x	C	A	1266	0.1667	1170	0.3188	<0.001
rs33961474	1000Genomes	delT	T	1006	0.2505	1008	0.2004	0.004
rs7725218	1000Genomes_30x	A	G	1266	0.3752	1170	0.3803	**0.413**
rs35372932	1000Genomes_30x	T	C	1266	0.0727	1170	0.3162	<0.001
rs1322599	1000Genomes_30x	T	C	1266	0.1927	1170	0.6205	<0.001
rs72838643	1000Genomes_30x	G	A	1266	0.1177	1170	0.1949	<0.001
rs2524079	1000Genomes_30x	A	G	1266	0.4605	1170	0.2564	<0.001
rs3134745	1000Genomes_30x	T	C	1266	0.2504	1170	0.1111	<0.001
rs78084314	1000Genomes_30x	T	C	1266	0.0308	1170	0.0205	**0.07**
rs445	1000Genomes_30x	T	C	1266	0.1224	1170	0.3624	<0.001
rs732330	1000Genomes_30x	C	T	1266	0.1943	1170	0.3513	<0.001
rs2915617	1000Genomes_30x	G	C	1266	0.7338	1170	0.8	<0.001
rs8180895	1000Genomes_30x	A	G	1266	0.0079	1170	0.0829	<0.001
rs13280978	1000Genomes_30x	T	C	1266	0.6572	1170	0.7726	<0.001
rs34092021	1000Genomes_30x	A	G	1266	0.0861	1170	0.2154	<0.001
rs12221430	1000Genomes_30x	G	C	1266	0.6335	1170	0.6752	0.017
rs139315440	1000Genomes_30x	A	G	1266	0.0261	1170	0.3359	<0.001
rs2419316	1000Genomes_30x	G	A	1266	0.0806	1170	0.3906	<0.001
rs7979874	1000Genomes_30x	T	C	1266	0.0158	1170	0.1179	<0.001
rs2040571	1000Genomes_30x	A	G	1266	0.0821	1170	0.2145	<0.001
rs8017228	1000Genomes_30x	C	A	1266	0.3831	1170	0.4393	0.003
rs4794822	1000Genomes_30x	T	C	1266	0.3791	1170	0.4487	<0.001
rs141236451	1000Genomes_30x	dupA	NA	1266	0.1524	1170	0.1855	0.017
rs117006166	1000Genomes_30x	A	G	1266	0.0498	1170	0.1487	<0.001
rs312068	1000Genomes_30x	C	T	1266	0.4905	1170	0.947	<0.001
rs3216949	1000Genomes_30x	dupC	NA	1266	0.8855	1170	0.6658	<0.001

Ref Allele signifies reference allele. Alt Allele signifies alternative allele. Alt Allele Fre indicates alternative allele frequency. NA means no frequency provided in the database.

The data on Ref Allele, Alt Allele and Sample Size were obtained from the dbSNP website (https://www.ncbi.nlm.nih.gov/snp/).

$^{+}$The statistical method used is Chi-square test.

Bold fonts indicate no statistically significant differences (*P*>0.05).

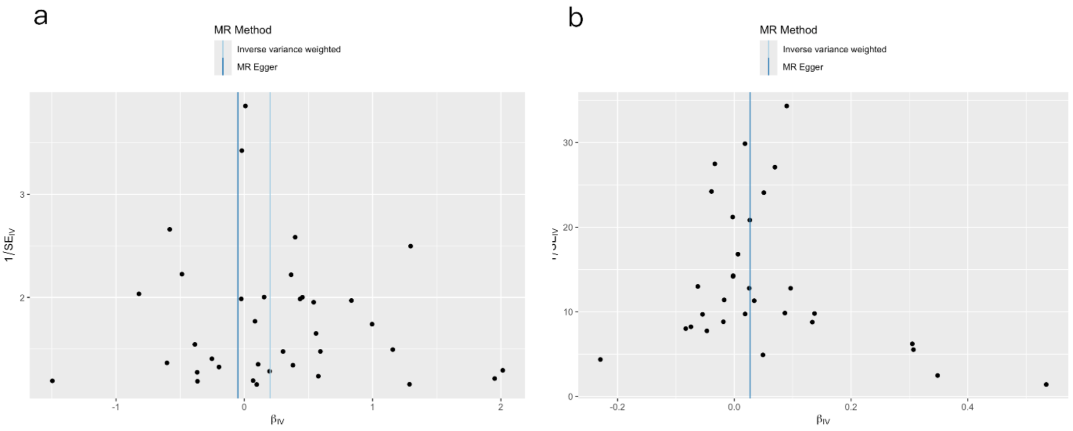

Figure S1. Funnel plot (a) in the East Asian population; (b) in the European population.
